# Supplementary material for: Genomic epidemiology of SARS-CoV-2 in Peru from 2020 to 2024
Source: Commun Med (Lond). 2026 Jan 9;6:22. doi: 10.1038/s43856-025-01273-z (PMC12789620; doi:10.1038/s43856-025-01273-z)
Supplement: Supplementary file 2 — Supplementary Information [file 43856_2025_1273_MOESM2_ESM.pdf]

SUPPLEMENTARY FIGURES

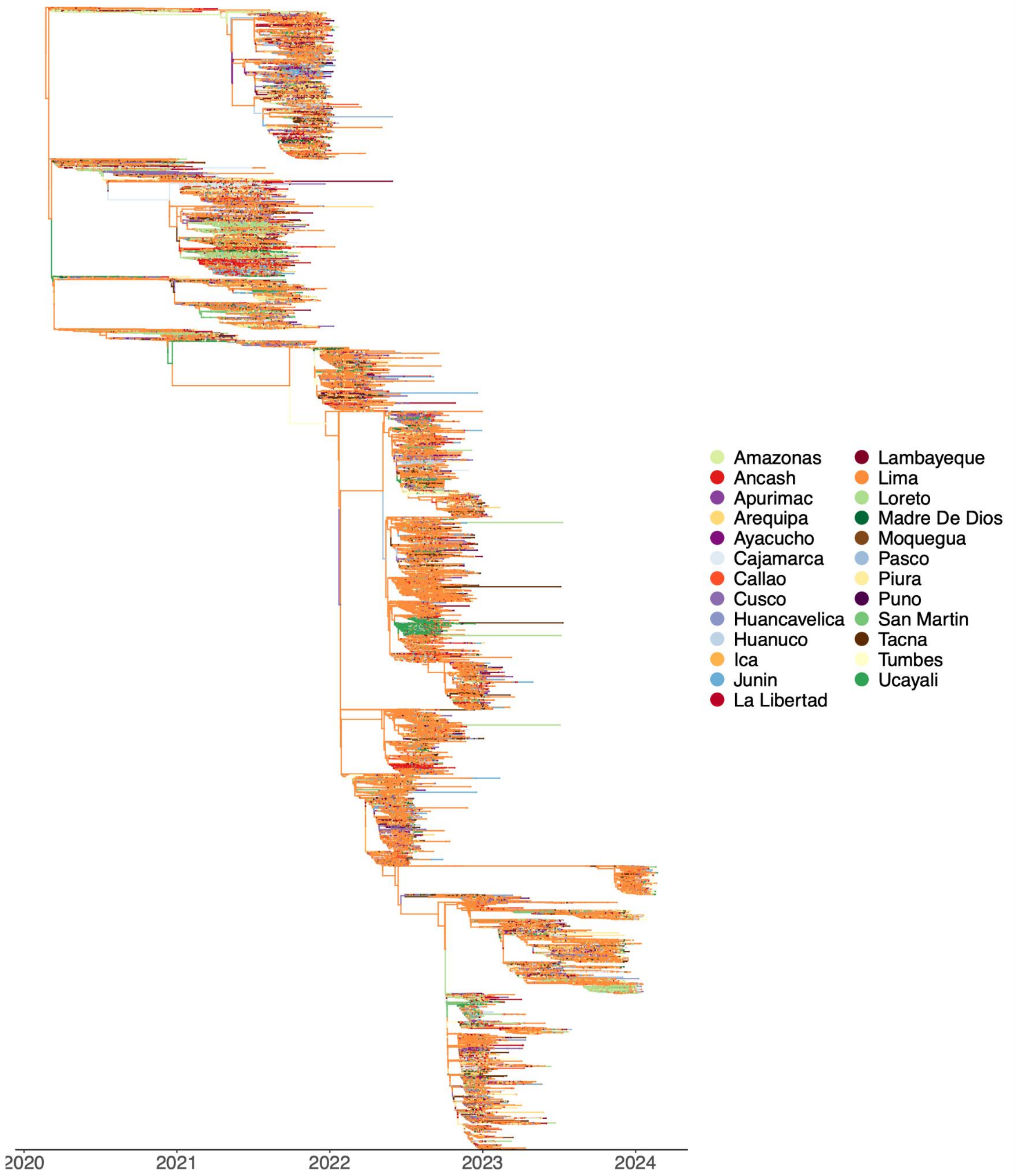

Supplementary Figure 1. A timed phylogeny of all SARS-CoV-2 sequences from Peru included in this study, with branches colored by region at the node, as inferred by the ancestral state reconstruction analysis.

a

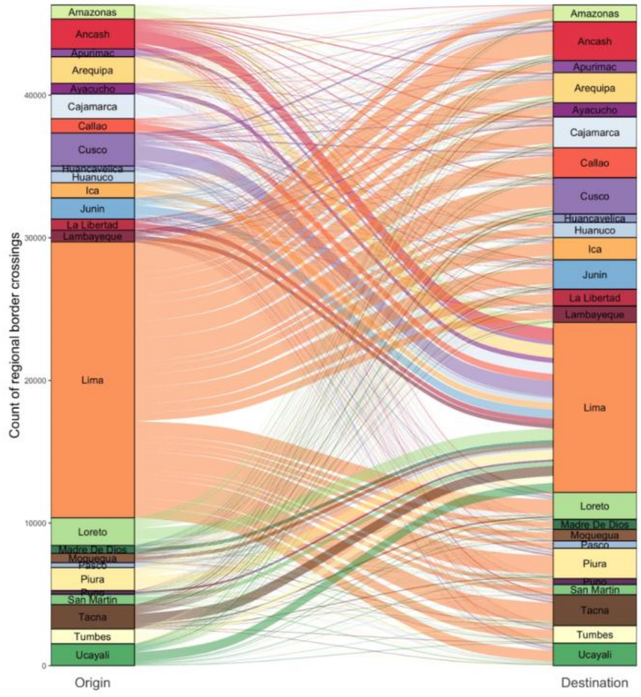

b

Region	Number of sequences (N = 43454)	Proportion of total sequences	Department population proportion
Lima	21067	0.42	0.32
Ancash	2350	0.05	0.04
Cusco	2092	0.04	0.04
Cajamarca	1801	0.04	0.05
Tacna	1790	0.04	0.01
Callao	1771	0.04	0.03
Piura	1763	0.04	0.06
Arequipa	1728	0.03	0.05
Junin	1722	0.03	0.04
Loreto	1654	0.03	0.03
Ucayali	1491	0.03	0.02
Ica	1250	0.03	0.03
Tumbes	998	0.02	0.01
La Libertad	976	0.02	0.06
Amazonas	972	0.02	0.01
Lambayeque	919	0.02	0.04
Huanuco	879	0.02	0.02
Ayacucho	784	0.02	0.02
Apurimac	680	0.01	0.01
Moquegua	648	0.01	0.01
San Martin	577	0.01	0.03
Madre De Dios	561	0.01	0.01
Huancavelica	486	0.01	0.01
Pasco	418	0.01	0.01
Puno	347	0.01	0.04

Supplementary Figure 2. (a) Inferred movements of SARS-CoV-2 infections from an internal node to a tip, representing a sequenced case, between the 25 departments of Peru. (b) Total number of sequences collected per region, the proportion of the total sequences collected in each region, and the proportion of the total population of Peru in each region.

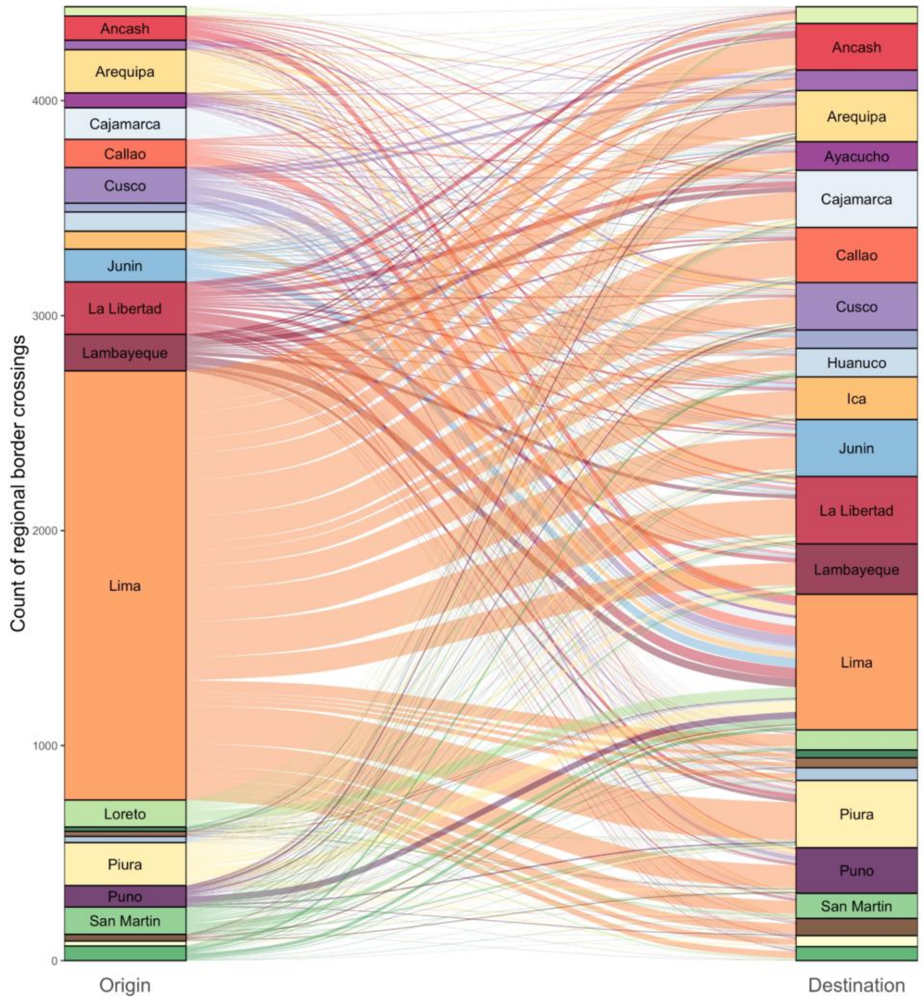

Supplementary Figure 3. Alluvial plot of movements between departments when only considering movements to the sampled tips from the node preceding it.

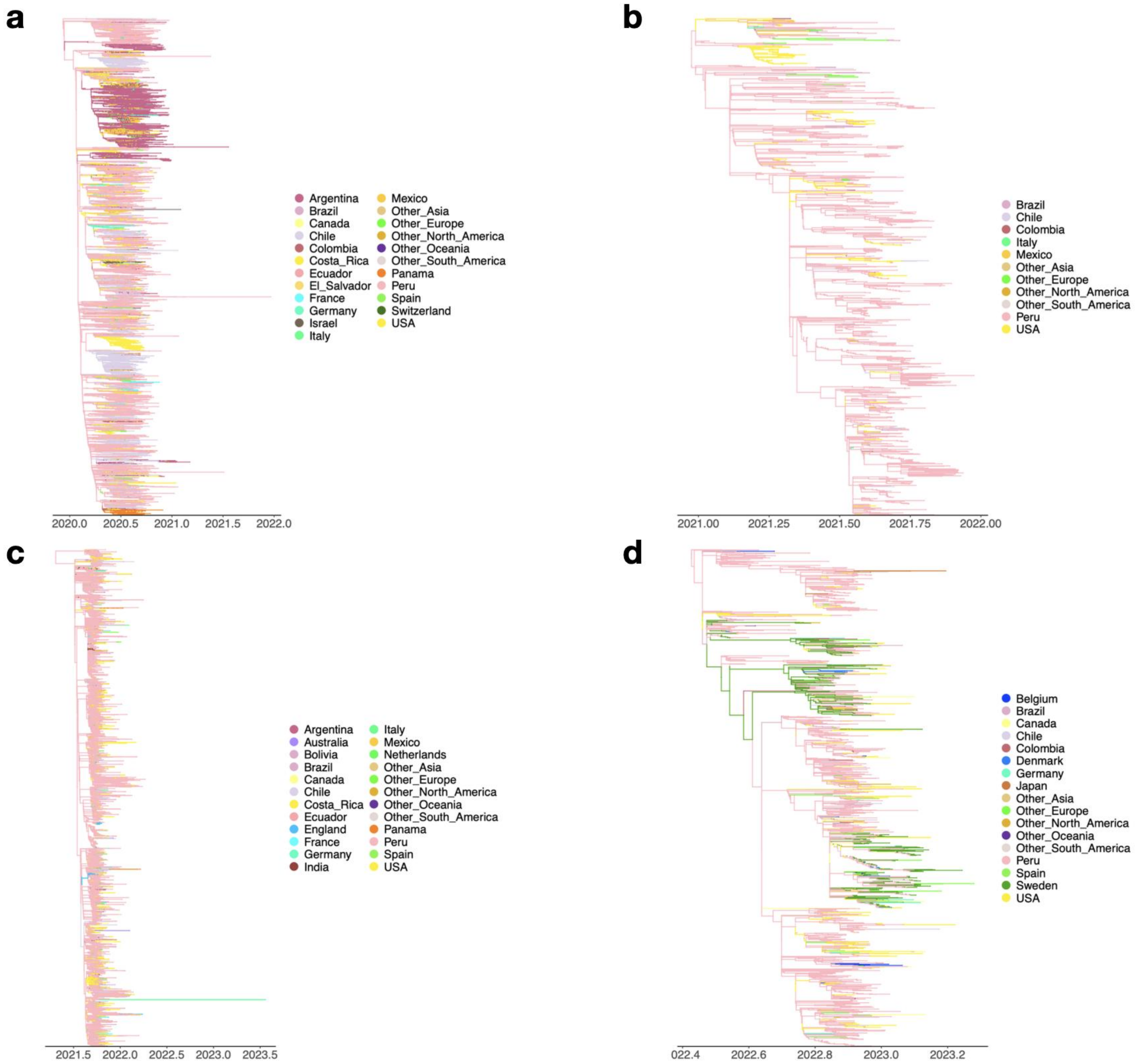

Supplementary Figure 4. Timed phylogenetic trees of all SARS-CoV-2 sequences of (a) Lambda C.37, (b) Gamma P.1.12, (c) Omicron XBB.2.6, and (d) Omicron DJ.1 variants, with branches colored by country of collection or inferred country state of the internal node.

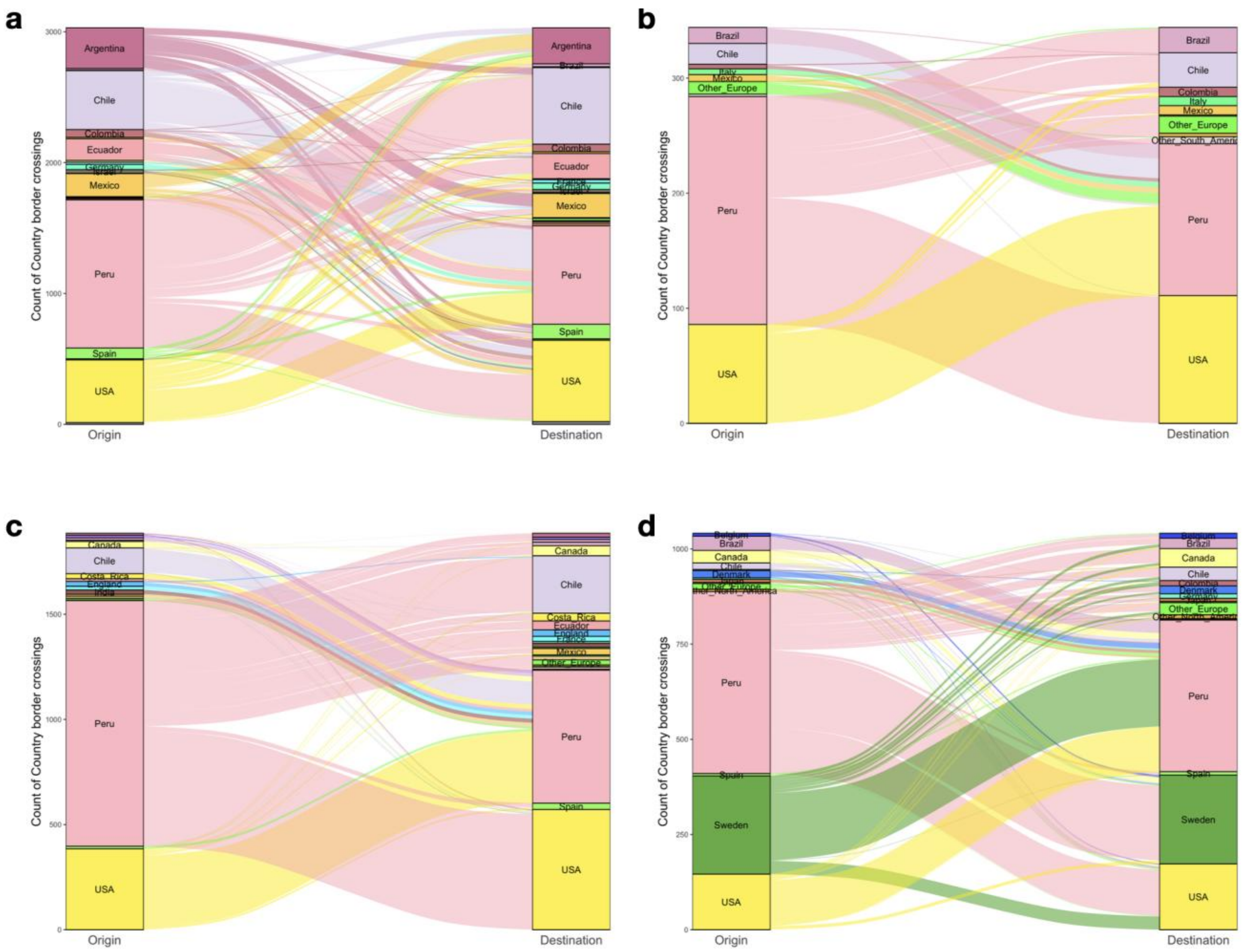

Supplementary Figure 5. Inferred movements between countries of sample collection for four sub-lineages with a Peruvian origin. (a) Lambda C.37, (b) Gamma P.1.12, (c) Omicron XBB.2.6, and (d) Omicron DJ.1 variants.

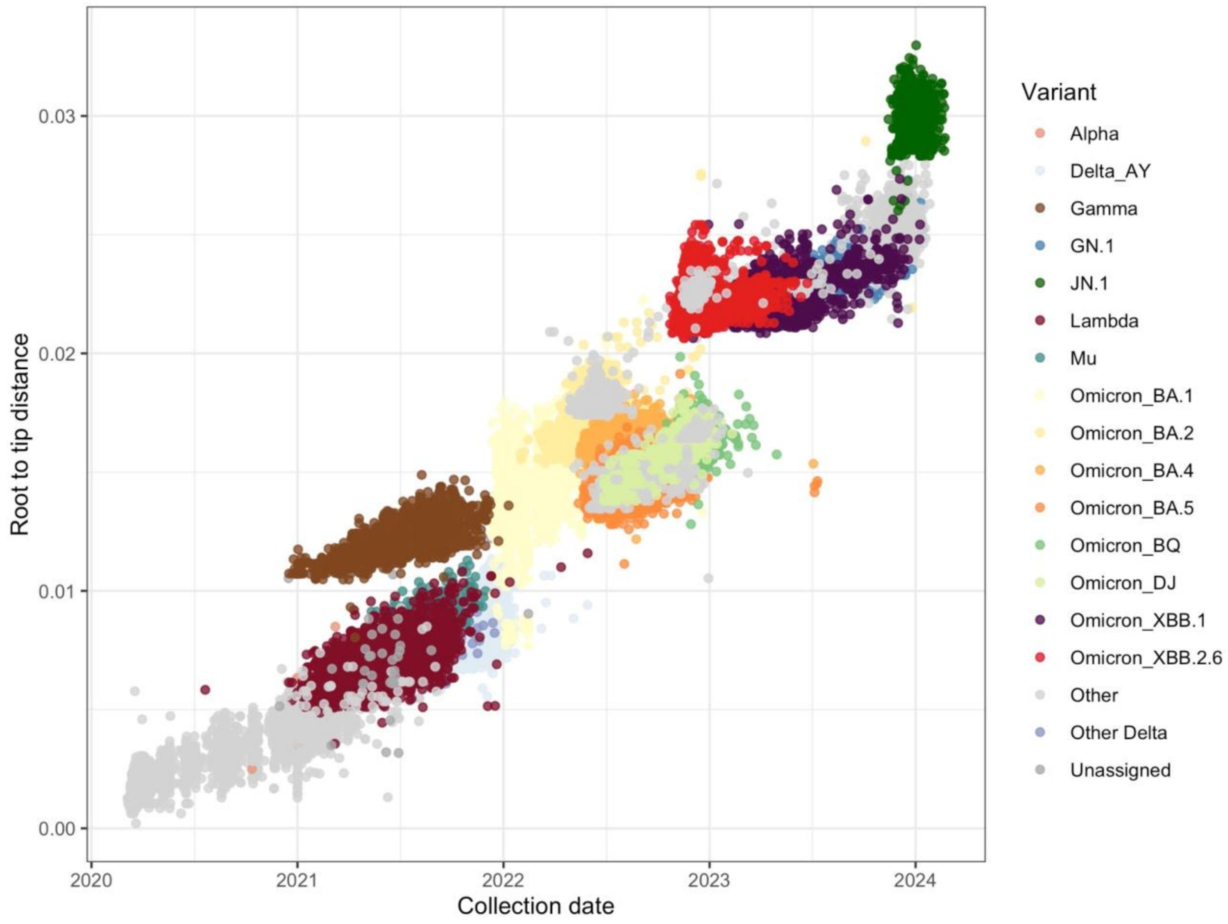

Supplementary Figure 6. Root-to-tip distance calculated using TempEst from the untimed maximum likelihood phylogeny of all Peruvian SARS-CoV-2 sequences included in the study, colored by variant (correlation coefficient=0.90, R2=0.81).
