# Supplementary material for: Genomic epidemiology of SARS-CoV-2 in Peru from 2020 to 2024
Source: Commun Med (Lond). 2026 Jan 9;6:22. doi: 10.1038/s43856-025-01273-z (PMC12789620; doi:10.1038/s43856-025-01273-z)
Supplement: Supplementary file 3 — Description of Additional Supplementary files [file 43856_2025_1273_MOESM3_ESM.pdf]

Description of Additional Supplementary Files

Supplementary Data 1- SARS-CoV-2 Pango lineages (n=548) identified from all the Peruvian genomes in our dataset and their frequencies.

Supplementary Data 2- GISAID identifiers of all the sequences included in our analysis: All Peruvian sequences (n=49724), Lamba C.37 (n=9908), Gamma P.1.12 (n=1204), Omicron XBB.2.6 (n=6703), Omicron DJ.1 (n=1726).

Supplementary Data 3- Complete list of mutations identified in a high proportion of Peruvian strains of the four sub-lineages of Peruvian origin (Lambda C.37, Gamma P.1.12, Omicron XBB.2.6, and Omicron DJ.1) that were found in a low proportion in all other strains collected in Peru.
